# Synthesis, characterization, and thermal and computational investigations of the l-histidine bis(fluoride) crystal

**DOI:** 10.1007/s00894-022-05168-x

**Published:** 2022-07-19

**Authors:** Ian Felipe Sousa Reis, Jailton Romão Viana, João Gomes de Oliveira Neto, Stanislav R. Stoyanov, José Walkimar de M. Carneiro, Mateus Ribeiro Lage, Adenilson Oliveira dos Santos

**Affiliations:** 1 Center for Social Sciences, Health and Technology, Federal University of Maranhão - UFMA, 65900-410 Imperatriz, MA Brazil; 2 Natural Resources Canada, © Her Majesty the Queen in Right of Canada, as represented by the Minister of Natural Resources Canada, CanmetENERGY Devon, Devon, Alberta 2021 Canada; 3 Inorganic Chemistry Department, Chemistry Institute – Universidade Federal Fluminense (UFF), Niteroi, RJ Brazil; 4 Federal University of Maranhão - UFMA, Campus Balsas, 65800-000 Balsas, MA Brazil

**Keywords:** Nonlinear optical materials, Amino acid, Crystal, X-ray diffraction, DFT

## Abstract

Nonlinear optical materials have been investigated recently due to their potential technological applications in information storage and communications. In this context, semi-organic crystals can effectively combine the desired nonlinear optical properties of amino acids with the promising mechanical and thermal properties of inorganic materials. In this work, we have synthesized and characterized a semi-organic crystal of the amino acid l-histidine and hydrofluoric acid and investigated the chemical interactions between the organic and inorganic moieties. The crystal of l-histidine bis(fluoride) has been produced by slow solvent evaporation and characterized by X-ray diffraction (XRD) crystallography and thermogravimetric and differential thermal analyses. The XRD conducted using the Rietveld method shows that the unit cell is orthorhombic with the P2_1_2_1_2 space group and contains four l-histidine bis(fluoride) units. Both differential thermal analysis and temperature-dependent XRD show that the crystals are thermally stable up to 191°C and do not undergo phase transition. The computational Hirshfeld surface analysis of the crystal structure reveals the main intermolecular interactions. Density functional theory has been employed to calculate the ionic interaction energy and electrostatic potential maps and confirm the spontaneity of ionic association at 191°C. The combined experimental and computational results show that the thermal stability of the semi-organic l-histidine bis(fluoride) crystal makes it suitable for nonlinear optical applications in optical sensing and communication systems.

**Graphical abstract:**

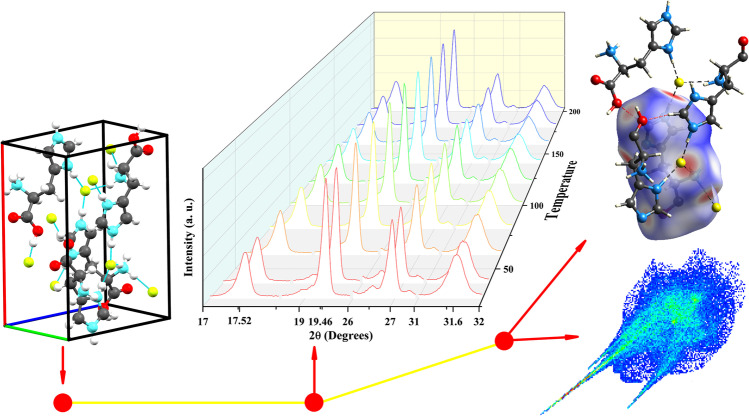

## Introduction

Second-order nonlinear optical materials (NLOs) have been the subject of several studies in recent decades due to their potential technological applications in optical communications, integrated optical systems, and information optical storage [1–3]. Currently, organic or inorganic optical crystals are being synthesized with a remarkable crystalline perfection [4]. However, each crystal class has certain advantages and disadvantages. On one hand, the purely organic crystals feature excellent NLO properties and a great birefringence, though they have a low thermal and mechanical stability [5]. On the other hand, inorganic materials have a high melting point, mechanical resistance, and chemical inertia; they have a low optical nonlinearity [6, 7]. In this context, new semi-organic compounds with properties of organic and inorganic crystals are being extensively explored, as they tend to combine the desired properties [8, 9]. In addition, a major advantage of most semi-organic materials is that upon forming ionic bonds, the resulting crystals acquire a higher probability of growth, greater thermal and mechanical resistance, and an increased chemical stability [6, 10–13].

Amino acids are bifunctional organic molecules that contain a carboxylic group (–COOH) and an amino group (–NH_2_). In α-amino acids, the characteristic side chain (*R*) of each amino acid is bonded to the same C atom (called alpha carbon, C_α_) as the amino and carboxylic groups. As a result of being bonded to four different groups, the C_α_ of all α-amino acids except glycine is chiral [14, 15]. Thus, α-amino acids acquire active optical properties due to being chiral species and directing the crystallization to non-centrosymmetric spatial groups. Moreover, the dipolar nature of amino acids provides unique physical and chemical properties, making them promising candidates for NLO applications [16, 17]. That is, the presence of the –COO^−^ and –NH_3_^+^ groups promotes the increase of the asymmetric polarizability of the organic material, providing asymmetry in the ground-state charge of the molecule, necessary for the development of second-order optical nonlinearity [12, 18–20].

Histidine is an essential α-amino acid, which can exist as two optical isomers, l-histidine and d-histidine. Only the l-isomer is bioactive. The l-histidine plays a vital role in several biological mechanisms, including the formation of hemoglobin and neurotransmitters in the brain and nervous system, and is essential for tissue growth and repair and glucose supply to the liver [21]. The l-histidine has a pKa close to neutrality and contains an imidazole side group [22–25].

The salts of l-histidine have attracted attention as promising materials for application in NLO after the verification that l-histidine tetrafluoroborate has better NLO properties than l-arginine phosphate monohydrate [19, 20, 26]. Since then, many researchers have synthesized and studied the properties of various crystals derived from this α-amino acid and proposed them as potential candidates for NLO applications [27, 28].

The l-histidine salt LHis·2HF has been synthesized for the first time in 1968 by Schmid using the reaction of l-histidine with hydrofluoric acid (HF) and water [29]. The synthesis of a semi-organic crystal of l-histidine hydrofluoric dihydrate and its NLO properties and the structure of a l-histidine bis(fluoride) crystal have also been reported [19, 26, 30]. The last salt contains delocalized π-electrons, electron donor, and acceptor groups in addition to fluoride ions strongly bonded to hydrogens in the organic moiety, conferring to its crystals high optical non-linearity and rapid optical response characteristics [30].

Electronic structure calculations have been widely used to study molecular systems and ionic associates with the aim of exploring their structural and thermodynamic properties [31]. Density functional theory (DFT) [32–34] is among the most extensively used model to explore NLO properties. For example, DFT has been used to investigate second-order NLO in l-histidine crystals, by determining the electrical dipole (μ), polarization (α), and hyperpolarization (β) of the crystal [35]. In addition, DFT has been employed to study chemical structure of crystals and for vibrational analysis [36–41].

A computational technique widely used to explore the intermolecular interactions between molecules in crystals is the Hirshfeld surface analysis [42]. Based on the description of atom-to-atom contacts, the Hirshfeld surface analysis provides an in-depth picture of how molecules come together in a crystalline structure [43, 44]. The Hirshfeld surface is a function of the sum of the electron density of atoms in a species divided by the sum of their closest neighbors, resulting in an isosurface that provides information about intermolecular interactions [45–47].

In this work, we synthesized l-histidine bis(fluoride) crystals by the slow evaporation method and characterized their structural and thermal properties by X-ray diffraction, thermogravimetric, and differential thermal analysis. The study is directed towards investigating the crystal’s structural and thermal stability at high temperatures, since these characterizations are important for the possible use of this material in NLO devices. In addition, computational studies using the DFT and Hirshfeld surface methods were conducted to understand the intermolecular interactions in the sample.

## Materials and methods

### LHis·2HF crystal synthesis

The crystals were synthesized by the slow evaporation method. Initially, a saturated aqueous solution of l-histidine (Sigma-Aldrich, purity ≤ 99%) and hydrofluoric acid (Impex, purity ≤ 48%) was prepared in a 1:2 mole ratio using deionized water. The solution was rotated on a shaker at 360 rpm for 2 h until complete homogenization and was then filtered through a filter paper with 25-μm pore. Subsequently, the solution was placed in a 50-mL beaker and covered with a plastic film, where several holes were drilled. Then, the covered beaker was placed in an oven with a controlled temperature of 35°C, favoring the slow evaporation of water and causing the supersaturation of the solution. The crystals were obtained after 37 days of storage.

### Characterization

The X-ray diffraction (XRD) measurements were performed with the crystals pulverized using an Empyrean PANanalytical diffractometer aligned with Bragg-Brentano reflection geometry (θ-θ), using Cu Kα radiation (λ = 1.5418 Å), operating at a voltage of 40 kV, and a current of 30 mA. The patterns were obtained at a step size of 0.02° for 3 s, and 2θ interval range from 5° to 45°. The high-temperature XRD measurements were performed using an Anton-Paar TTK 450 chamber coupled with the diffractometer. The sample diffraction pattern was measured in the temperature range from 30 to 190 °C. Rietveld refinement using the GSAS-EXPGUI interface [48] was used to analyze the crystal XRD data. From the obtained values of the lattice parameters at various temperatures, it was possible to calculate the linear coefficients of thermal expansion for each crystallographic direction using the following linear functions:

1 $${\alpha }_{\left[100\right]}=\frac{1}{a} \left(\frac{da}{dT}\right),$$

2 $${\alpha }_{\left[010\right]}=\frac{1}{b} \left(\frac{db}{dT}\right),$$

3 $${\alpha }_{\left[001\right]} =\frac{1}{c} \left(\frac{dc}{dT}\right),$$

where ${\alpha }_{\left[100\right]}$, ${\alpha }_{\left[010\right]}$, and ${\alpha }_{\left[001\right]}$ are the coefficients of thermal expansion; *da*, *db*, and *dc* are the variations of the lattice parameters, and *dT* is the temperature variation [49].

### Thermal analysis

Thermal gravimetric analysis (TGA) and differential thermal analysis (DTA) were used to observe the variation in mass and differential thermal flow, respectively, of the sample when exposed to a controlled temperature increase, to identify events such as melting point, structural phase transition, and decomposition. The TGA and DTA measurements were carried out simultaneously in a Shimadzu DTG-60 thermogravimetric analyzer with an α-alumina open crucible in a nitrogen atmosphere at a flow rate of 100 mL∙min^−1^, in the temperature range from 30 to 500 °C, with a heating rate of 10 °C∙min^−1^.

### DFT study

The DFT calculations were performed using the hybrid functional *ω*B97x-D with both dispersion and long-range interaction corrections, the 6-311++G(d,p) basis set, and the integral equation formalism version of the polarizable continuum model (IEF-PCM) of solvation, using water as the solvent (Ɛ = 78.4), as implemented in the Gaussian 16 software [50–52]. The DFT calculations include full geometry optimization, followed by vibrational analysis to confirm the optimized geometries as minima on the potential energy surface. The initial geometry of the main ionic associate containing 3 protonated histidine cations, connected by hydrogen bond to a fluoride anion, was taken from the XRD structure published by Petrosyan et al. [26]. This ionic associate was used as a representative block of the crystal. The charge and multiplicity for the whole associated system of [3LHisH_2_·F]^5+^ were defined as 5 and 1, respectively. Finally, the DFT results were analyzed with the Chemcraft software to visualize the optimized geometry and properties of the systems [53, 54].

The fluoride and protonated histidine ions were also studied individually. Then, variation of Gibbs free energy (Δ*G*), enthalpy (Δ*H*), entropy (Δ*S*), and total electronic energy corrected with ZPVE (Δ*E*) associated to the ionic association were calculated based on Eqs. 4, 5, 6, 7:

4 $$\Delta G={G}_{T}-\left(3.{G}_{H}+{G}_{F}\right)$$

5 $$\Delta H={H}_{T}-\left(3.{H}_{H}+{H}_{F}\right)$$

6 $$\Delta G=\Delta H-T.\Delta S$$

7 $$\Delta E={E}_{T}-\left(3.{E}_{H}+{E}_{F}\right)$$

where ${G}_{T}$, ${G}_{H}$, and ${G}_{F}$ denoted the Gibbs free energies of the ionic associate, protonated histidine, and fluoride anion, respectively; ${H}_{T}$, ${H}_{H}$, and ${H}_{F}$ denoted the enthalpies of the ionic associate, protonated histidine, and fluoride anion, respectively; *E*_*T*_, *E*_*H*_, and *E*_*F*_ were the total electronic energies corrected with the zero-point vibrational energy (ZVPE) of the ionic associate, protonated histidine, and fluoride anion, respectively. All thermodynamic quantities were calculated at 298.15 K and 464.15 K, both at 1 atm. Basis set superposition error (BSSE) was included in the calculation of the ionic association energies.

### Hirshfeld surface

The Hirshfeld surfaces and two-dimensional (2D) fingerprint graphics were obtained using the Crystal Explorer 17 software [55], to enable a detailed analysis of the interactions between the chemical species in the crystal. The Hirshfeld surfaces were mapped as a function of normalized distance (*d*_norm_), to provide three-dimensional (3D) representations of close contacts, defined in terms of distances from a given point on the surface to the nearest external (*d*_e_) and internal (*d*_i_) atom, and van der Waals radius (*r*_vdW_). The mapped Hirshfeld surfaces were rendered through a red-white-blue color scheme, where red was used for close contacts, white was used for contacts near the van der Waals radius, and blue was used for long distance contacts [45, 47]. The 2D graphs, given as functions of *d*_e_ and *d*_i_, cover all intermolecular contacts, quantify specific interactions, and summarize the frequency of each combination [43, 44].

## Results and discussion

### X-ray diffractometry

**Fig. 1:**
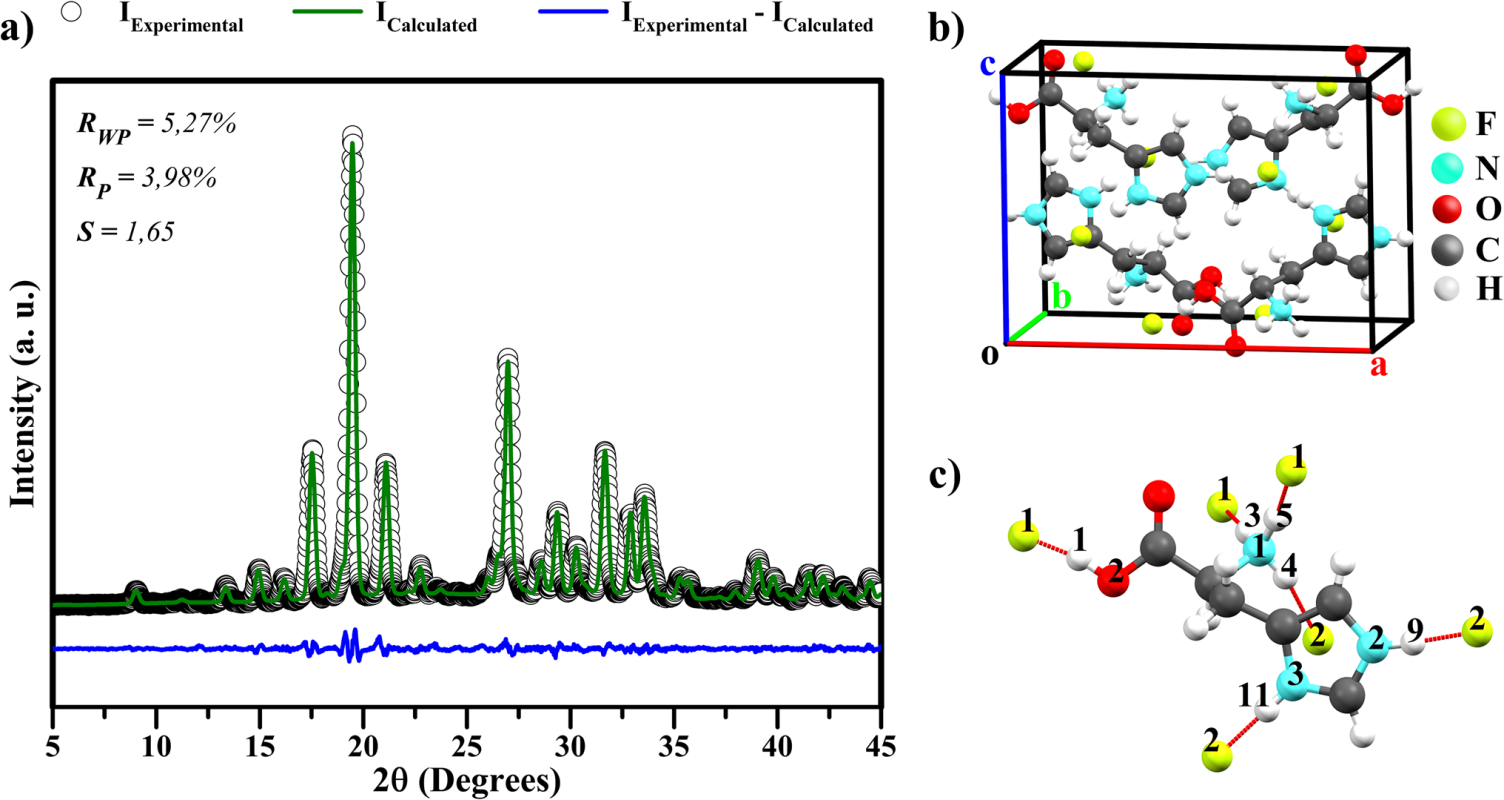
**a** XRD Rietveld refinement of LHis·2HF crystal at room temperature; (**b**) unit cell of LHis·2HF; and (**c**) molecular structure of LHis·2HF, showing the hydrogen bonds as red sticks

Figure 1(a) shows the Rietveld refinement of the LHis**·**2HF XRD pattern at room temperature. The Rietveld refinement *R*-factors for the weighted profile (*R*_WP_) and residual of least-squares refinement (*R*_P_) are 5.27% and 3.98%, respectively. The goodness of the fit indicator *S* is 1.65. These values indicate that the Rietveld refinement results obtained for the crystal have a high reliability [56].

The LHis**·**2HF at room temperature crystallizes in an orthorhombic phase (P2_1_2_1_2-space group) with four molecules per unit cell (Fig. 1(b)) and has the following refined lattice parameters: *a* = 13,123(1) Å, *b* = 6.5810(0) Å, *c* = 9.651(5) Å, α = β = γ = 90°, and *V* = 833.499 Å^3^. These parameters are in agreement with those reported by Petrosyan et al. [26], with a percentage difference of less than 0.5%.

Figure 1(c) shows the LHis**·**2HF structure with its hydrogen bonds that are essential to maintain the material’s structural stability. According to Petrosyan et al. [26], LHis**·**2HF has a different hydrogen bonding scheme from LHis**·**2HCl and LHis**·**2HBr. There is a strong hydrogen bond between the acidic hydrogen atom of the carboxylic group of the α-amino acid and one of the fluoride ions: O2–H1**···**F1. Hydrogen bonds also form between the N–H proton of imidazole (when protonated) and the second fluoride ion: N2–H9**···**F2 and N3–H11**···**F2. When protonated (as –NH_3_^+^), the amino group also forms two hydrogen bonds with F1 and F2, namely N1–H3**···**F1, N1–H5**···**F1, and N1–H4**···**F2. Thus, each –NH_3_^+^ cation forms three hydrogen bonds.

### Thermal analysis (TGA-DTA)

**Fig. 2:**
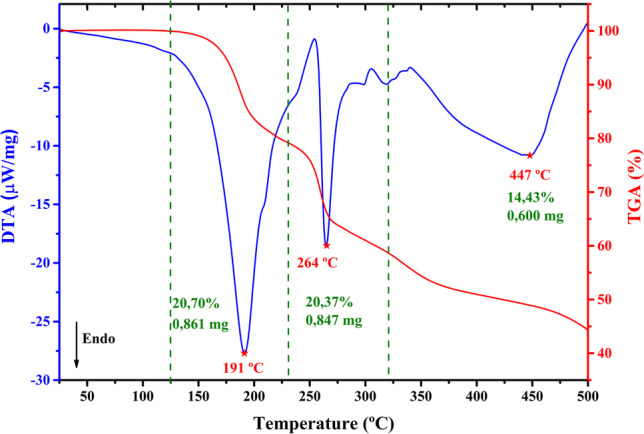
TGA-DTA curves of the LHis·2HF crystal

The LHis**·**2HF crystal thermogram is shown in Fig. 2. Analyzing the DTA curve, an endothermic peak is observed between 125 and 230 °C that was associated to the first weight loss (I) of approximately 20.70% in the TGA curve, equivalent to 0.861 mg of the initial crystal mass. This event is followed by two more stages of mass loss, i.e., (II) 231 to 320 °C, where an endothermic peak appears at 264 °C on the DTA curve, with a weight loss of 20.37% (0.847 mg) in the TG curve, and (III) 320 to 500 ºC, shows a weight loss of 14.43% (0.600 mg). These events are attributed to the decomposition of the LHis**·**2HF crystal.

From the TGA-DTA curve, it was observed that the LHis**·**2HF crystal is thermally stable up to 191 °C, without any transformation or transition in this temperature range (Fig. 2). Dhanuskodi and Ramajothi (2004) have performed a thermal and optical study of the crystal of l-histidine tetrafluoroborate [57] and also observed a good thermal stability up to its melting point of 235 °C. On the other hand, Madhavan et al. [19] have carried out DTA studies of the l-histidine hydrofluoride dihydrate crystal and revealed that the material showed thermal stability only up to 108 °C, due to the loss of lattice water. The value found by Madhavan et al. is lower than the one observed in our work. Thus, the good thermal stability of the LHis**·**2HF crystal can be attributed to the hydrogen bonding scheme, in conjunction with the anhydrous structure of the material, as rationalized by Faria et al. for LHis**·**HCl**·**H_2_O [24]. Thus, based on its thermal stability, LHis**·**2HF can be utilized for optoelectronic applications up to 191 °C.

### XRD at high temperatures

**Fig. 3:**
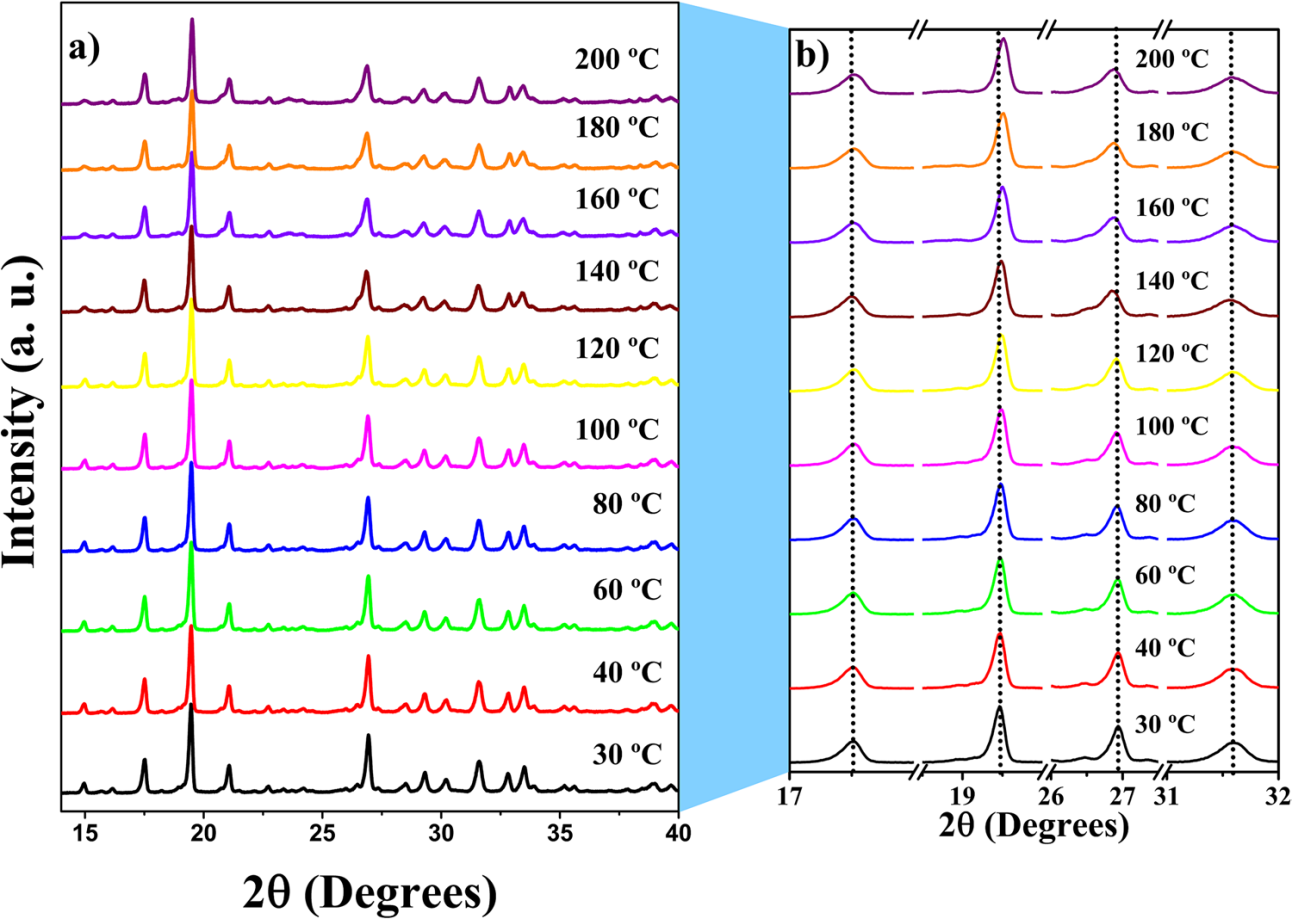
Diffraction patterns of the LHis·2HF crystal as a function of temperature (**a**) 15° and 40°, (**b**) 17° and 18°; 18.5° and 20°; 26° and 27.5°; 31° and 32°

The TGA-DTA results for LHis**·**2HF crystal show no evidence of the physical phenomenon of phase transition in the studied temperature range. To confirm the stability of the crystal, its thermal and structural behavior were also analyzed by using XRD at high temperatures. The crystal was subjected to XRD in the range from 30 to 190 °C, where the first decomposition event was detected by thermal analysis. The diffraction patterns are shown in Fig. 3.

The diffraction patterns obtained between 30 and 190 °C show the LHis**·**2HF crystal only in an orthorhombic phase. In the diffractograms in Fig. 3(a), only small perceptible changes associated with the minimal structural changes in the crystal as a function of the temperature change are observed. In Fig. 3(b), it is possible to notice that some of the crystallographic axes undergo expansion or contraction with the sample’s gradual heating. The diffraction pattern undergoes a small displacement of the Bragg peaks to the left or to the right, accompanied by intensity loss at higher temperatures. This can be seen when following the dotted lines in Fig. 3(b) in the diffraction peaks with 2θ of 17.52°, 19.46°, 26.94°, and 31.60°.

**Fig. 4:**
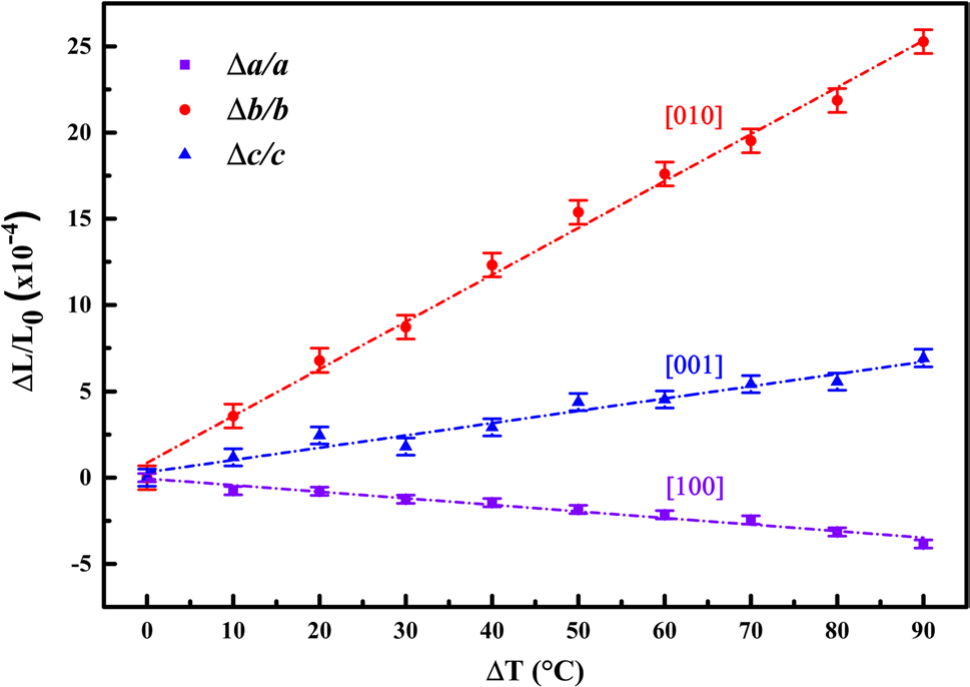
Thermal expansion coefficient of the LHis·2HF crystal up to 100 °C.

After obtaining the lattice parameters as a function of the temperature, it was possible to estimate the crystal thermal expansion coefficients at temperature between 30 and 120 °C, using Eqs. 1, 2, and 3. Figure 4 shows the variation of the lattice parameters (Δ*L*/*L*_0_) as a function of the temperature, where the thermal expansion coefficient is calculated from the slope of each line, obtained as a result of a linear fit of all points. The calculated slopes are $\alpha_{\left[100\right]}$ = –3.80(7) × 10^–6^ C^**−**1^, $\alpha_{\left[010\right]}$ = 27.20(6) × 10^–6^ C^**−**1^, and $\alpha_{\left[001\right]}$ = 7.13(3) × 10^–6^ C^**−**1^. These results show that the thermal expansion is negative along axis *a* and positive along axes *b* and *c*. Thus, the LHis**·**2HF crystal clearly exhibits anisotropic behavior, which may be related to the spatial orientation of the hydrogen bonds in the lattice, as reported for LHis**·**HBr**·**H_2_O [58].

### DFT study

**Fig. 5:**
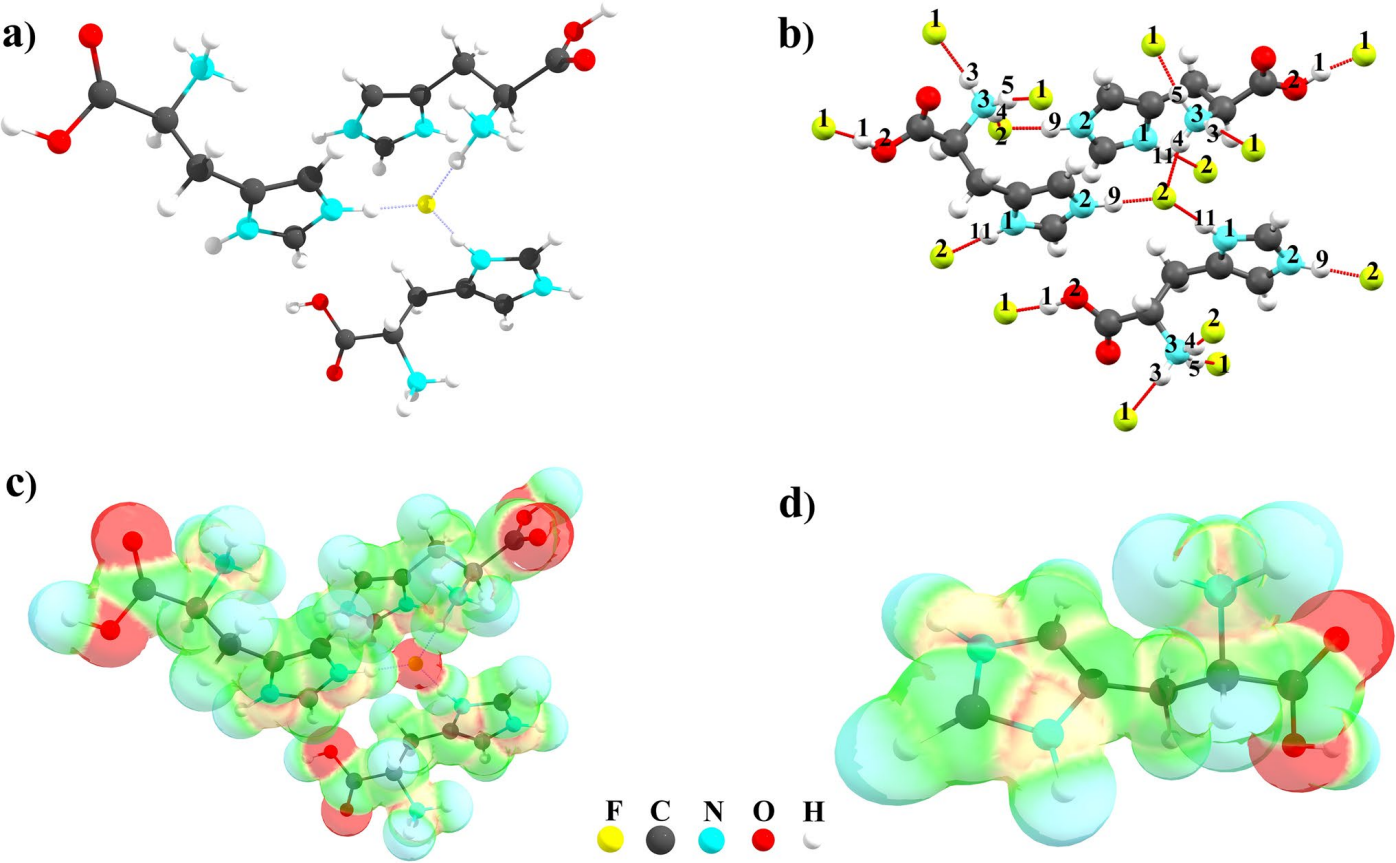
**a** Optimized structure of the [3LHisH_2_·F]^5+^ associate; (**b**) experimental structure collected from the crystallographic database; (**c**) electrostatic potential of [3LHisH_2_·F]^5+^ mapped on the electron density surface; (**d**) electrostatic potential map

The geometries of the main ionic associate present in the crystal have been fully optimized by the means of DFT, using the *ω*B97x-D functional, 6-311++G(d, p) basis set, and IEF-PCM solvation method. The optimized geometries are confirmed as true minima on the potential energy surfaces by calculating the vibrational frequencies (confirmed by the presence of positive values only). Selected optimized structures are shown in Fig. 5(a). In the ionic associate, hydrogen bonds between the hydrogen atoms of the NH_2_ groups and the fluoride anion (F^–^) are observed in different regions of the protonated histidine. The results show that angles and interatomic distances converged to values close to those found experimentally in the crystal. Petrosyan et al. [26] reported that the N(imidazole)–F(fluoride) distances are 2.5421(16) Å and 2.5269(15) Å, and that the dihedral angle between the main chain carbon atoms of imidazole is −171.85(12)°. These values compare favorably with the optimized N(imidazole)–F(fluoride) distances of 2.5721 Å and 2.5685 Å and the imidazole dihedral angle of −179.09°.

The analysis based on Eqs. 4–7 yields the ionic association Gibbs free energy, enthalpy, entropy, and electronic energy corrected with ZPVE values of −20.34 kcal∙mol^**−**1^, −52.59 kcal∙mol^**−**1^, −0.11 kcal∙mol^**−**1^∙K^**−**1^, and −52.98 kcal∙mol^**−**1^, respectively, at 298.15 K. After BSSE correction, the ionic association electronic energy corrected with ZPVE is −48.21 kcal∙mol^**−**1^. The values of Gibbs free energy, enthalpy, and electronic energy corrected with ZPVE and BSSE are negative. These parameters indicate that the formation of the ionic associate is exothermic and spontaneous, at room temperature. The spontaneous association is attributed to the formation of a network of strong hydrogen bonds between the protonated histidine and the fluoride anion [31, 59, 60].

The thermochemical analysis is also conducted at a temperature of 464.15 K. The calculated values of Gibbs free energy, enthalpy, and entropy are −2.63 kcal∙mol^**−**1^, −51.56 kcal∙mol^**−**1^, and 0.11 kcal∙mol^**−**1^∙K^**−**1^, respectively. When comparing the thermochemical results, it is observed that the Gibbs free energy undergoes a significant increase, as the temperature is increased from 298.15 to 464.15 K, but remains negative. The enthalpy and entropy undergo less significant changes. Our results also indicate that the increase in temperature favors an increase in the entropy of the system, which is related to an increase in the Gibbs free energy. The negative value of the Gibbs free energy of ionic association at 464.15 K (191°C) corroborate the experimental TGA-DTA findings that the material would be stable at this higher temperature.

The electrostatic potential of the ionic associate was mapped on the electron density isosurface (shown in Fig. 5(c)). The largest localization of negative charges, shown in red, are localized near the fluoride anion and the oxygen atoms of the carboxylate groups. The electrostatic potential map of the protonated l-histidine (Fig. 5(d)) shows the regions of positive electrostatic potential (in blue), which indicate favorable positions for nucleophilic attack, as well as regions with negative electrostatic potentials (in red), which indicate areas more favorable to electrophilic attack. In this case, the most positive potential is found near the –NH_3_^+^ groups, and the most negative potential near the carboxylic oxygen atoms.

### Hirshfeld surface analysis

**Fig. 6:**
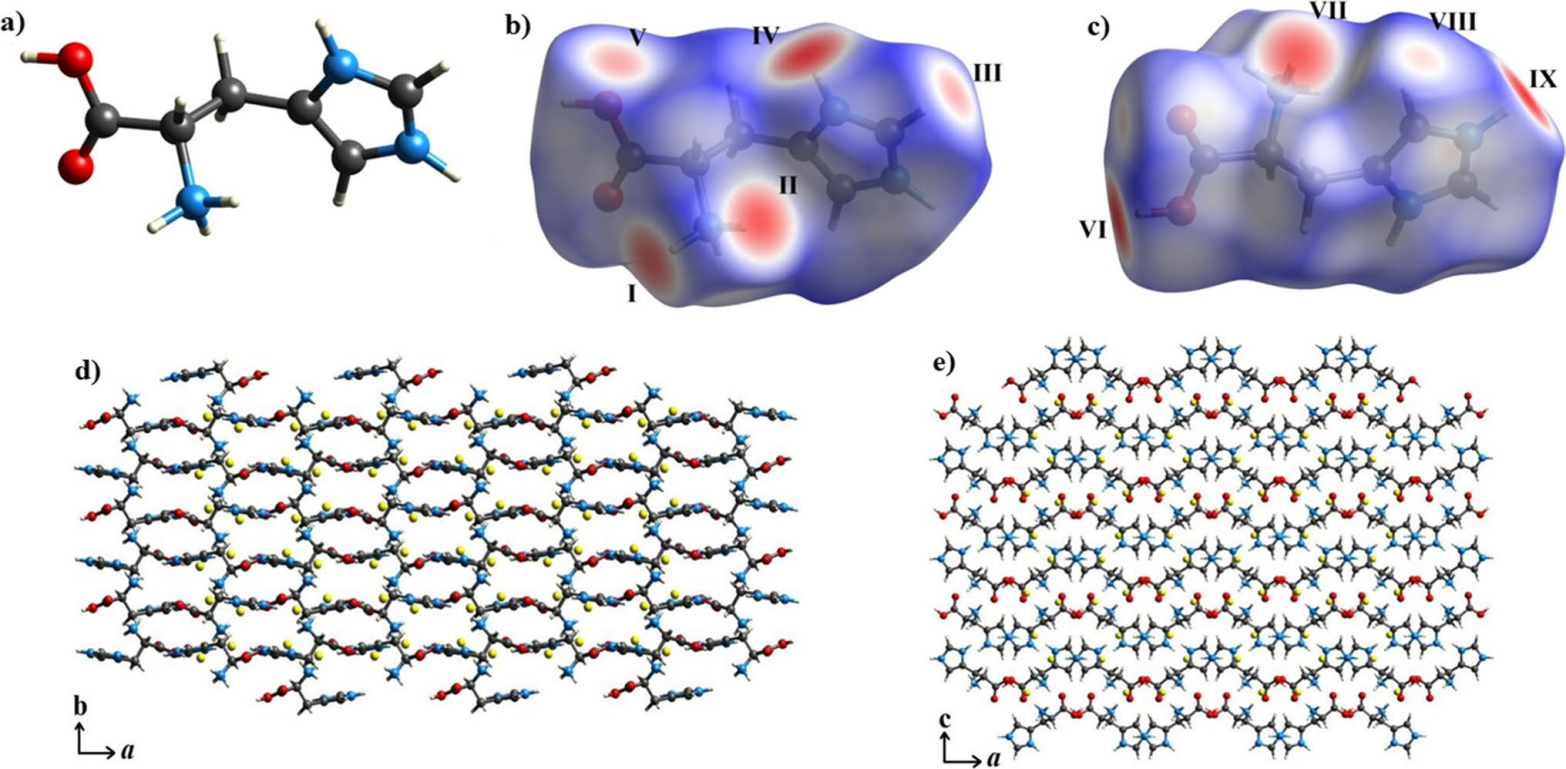
**a** Structure of l-histidine protonated in the –NH_3_^+^ group; **b** Hirshfeld surface of protonated l-histidine mapped according to *d*_norm_; **c** Hirshfeld surface rotated by 180º relative to **a** around the horizontal axis; **d** expanded LHis·2HF crystal structure along the a and b axes; and **e** expanded LHis·2HF crystal structure along the a and c axes

To better understand the intermolecular interactions in the LHis**·**2HF crystal, Hirshfeld surfaces were produced and analyzed with respect to the protonated histidine molecule, shown in Fig. 6(a). The strongest interactions can be seen in Fig. 6(b and c)  as red regions, corresponding to the strongest hydrogen bonding sites. The interactions labeled as I, II, and VII correspond to the hydrogen bonds with F1 and F2, i.e., N1–H3**···**F1, N1–H5**···**F1 and N1–H4**···**F2 of the amino group, when protonated as –NH_3_^+^. Interactions IV and IX are those with the hydrogen bonds involving the imidazole N–H group through bonds N2–H9**···**F2 and N3–H11**···**F2 (atom labels are according to the notation of Fig. 1(c)). The hydrogen bond with the carboxylic group of the amino acid with one of the fluorine ions (O2–H1**···**F1) is shown in interaction VI. All the interactions mentioned agree with the XRD measurements, shown in Fig. 1(c). Interactions III, V, and VIII, indicated by white shading, are related to contacts with another protonated histidine in the neighborhood. When the crystalline lattice is expanded, a “beehive” structure is observed when seeing along the ab plane (Fig. 6(d)). When the crystal structure is rotated 90°, the orthorhombic structure is observed along the ac plane (see Fig. 6(e)). It is important to note that these Hirshfeld surfaces show intermolecular interactions that have not previously been reported in the literature.

The 2D fingerprint plot provides a histogram as a function of the colored surface points fraction, in which the blue color represents few points and the red color represents many points, accounting for specific close contacts [43, 44, 47]. The calculation provides a quantitative information on the intermolecular interactions, which makes clearer the analysis of the interactions between the chemical species in the crystal.

**Fig. 7:**
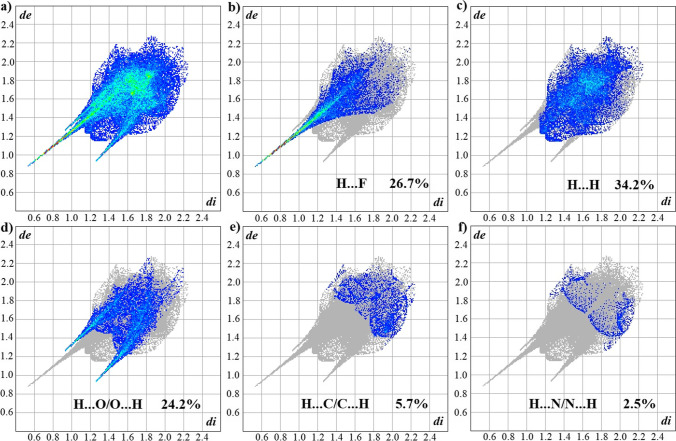
**a** Full 2D fingerprint graph of l-histidine protonated in –NH_3_^+^ and interaction-specific fingerprint graphs **b** H···F, **c** H···H, **d** H···O/O···H, **e** H···C/C···H and H···N/N···H

The cumulative 2D fingerprint graph is shown in Fig. 7(a). The decomposed graphs of the H**···**F, H**···**H, H**···**O/O**···**H, H**···**C/C**···**H, and H**···**N/N**···**H interactions, with their respective percentage contributions, are shown in Fig. 7(b, c, d, e, and f) , respectively. The fingerprint graphs (Fig. 7(b–f)) correspond to the most important interactions, contributing to 93.3% to the total Hirshfeld surface. The remaining percentages are related to less significant interactions. In 2D fingerprint graphics, the presence of long, thin peaks in the regions of lower *d*_e_ + *d*_i_ values indicates the presence of strong interactions on the surfaces [61]. Long, thin peaks are seen in Fig. 7(b and d) , corresponding to the H**···**F and H**···**O/O**···**H interactions, confirming these as the strongest interactions in the crystal (Fig. 7(c)). Additionally, the H**···**H interaction is the most representative in terms of percentage contribution to interactions between atoms.

**Fig. 8:**
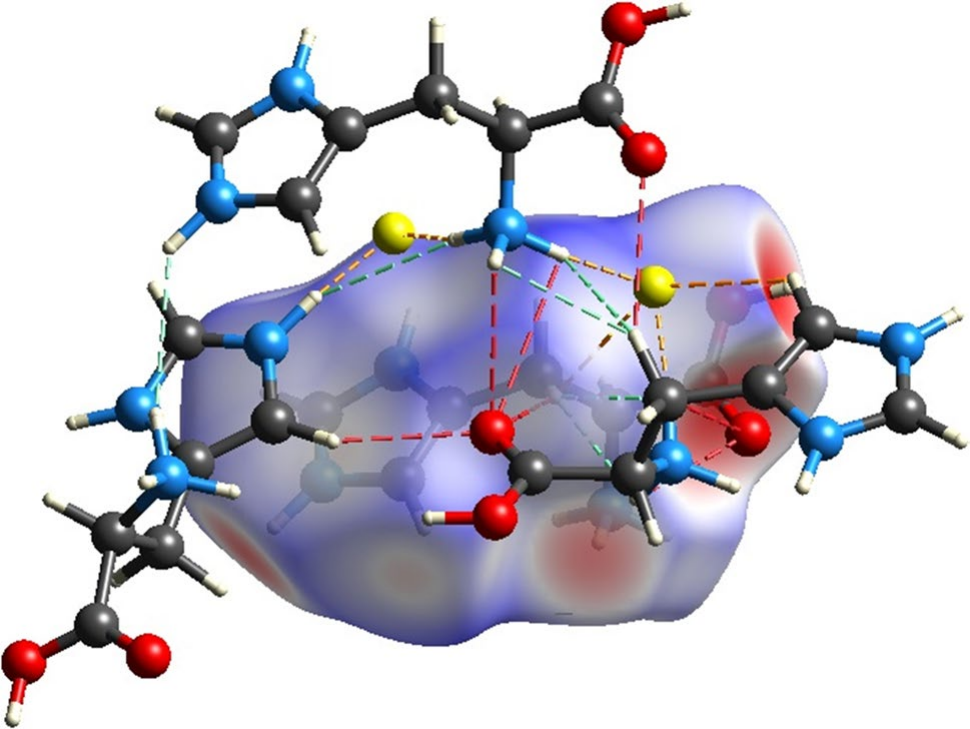
Intermolecular interactions of LHis·2HF crystal: dashed orange (H···F), red (H···O/O···H), and green lines (H···H)

Figure 8 shows a more detailed model of the intermolecular contacts, showing the three most significant interactions of the analyzed Hirshfeld surfaces of the LHis**·**2HF crystal. The dashed orange, red, and green lines show the H**···**F, H**···**O/O**···**H, and H**···**H contacts, respectively. Thus, Fig. 8 corroborates the findings presented in Fig. 6(b and c) , showing that the main intermolecular interactions involve hydrogen bonding, confirming their substantial contributions to the stability of the crystal.

## Conclusions

In this work, LHis·2HF crystals were successfully synthesized by the slow evaporation technique and their thermal stability was investigated by using high-temperature XRD and thermal analysis with simultaneous TGA and DTA measurements. The TGA-DTA analyses showed that the l-histidine bis(fluoride) crystal was thermally stable up to the range of 191 °C. No events were observed in the DTA curve related to phase transitions, what was confirmed by the XRD analysis as a function of temperature. The effect of temperature on the crystallographic axes of the unit cell was verified in the orthorhombic phase, where a markedly anisotropic behavior was observed. These studies made it possible to estimate the thermal expansion coefficients of the crystal. The thermal stability of the crystal can be related to the hydrogen bonding and the lack of water in its matrix.

Moreover, DFT computational studies and Hirshfeld surface analysis were conducted to investigate the intermolecular hydrogen bonding interactions. In the DFT study, we obtained the optimized geometry of the main ionic associate in the crystal. The thermodynamic results confirmed the spontaneous formation of the ionic associate at temperature as high as 191°C, as a consequence of the occurrence of a strong attraction between the ions in this system, even in water. In the solid state, strong attraction between the ions can also be expected, which justifies the high stability of the solid.

The Hirshfeld surfaces identified the main points of intermolecular interactions in the structure. The Hirshfeld fingerprint graphs provided quantitative insights on the frequency of the occurrence of the different close contacts. The main points of intermolecular interactions match with the interactions indicated in the DFT study.

Taken together, the crystal characterization, thermal stability, and computational studies show that LHis·2HF crystal exhibits the thermal properties and possesses the intermolecular hydrogen bonding interactions that make it suitable for NLO applications. Also, these results contribute to developing a comprehensive understanding of the stabilization of semi-organic crystals with attractive NLO properties.

## Funding 

This research was supported by Fundação de Amparo à Pesquisa e ao Desenvolvimento Científico e Tecnológico do Maranhão (FAPEMA), Coordenação de Aperfeiçoamento de Pessoal de Nível Superior (CAPES), and Conselho Nacional de Desenvolvimento Científico e Tecnológico (CNPq) and the Program of Energy Research and Development (PERD).

## Data Availability

Data and materials are available on request from the authors.

## Code availability

This work was conducted using the commercial software Gaussian 16, Chemcraft, and the freeware Crystal Explorer 17.
